# *In Vivo* Evaluation of the Acute Pulmonary Response to Poractant Alfa and Bovactant Treatments in Lung-Lavaged Adult Rabbits and in Preterm Lambs with Respiratory Distress Syndrome

**DOI:** 10.3389/fped.2017.00186

**Published:** 2017-08-31

**Authors:** Francesca Ricci, Fabrizio Salomone, Elke Kuypers, Daan Ophelders, Maria Nikiforou, Monique Willems, Tobias Krieger, Xabier Murgia, Matthias Hütten, Boris W. Kramer, Federico Bianco

**Affiliations:** ^1^Department of Preclinical Pharmacology, R&D, Chiesi Farmaceutici S.p.A., Parma, Italy; ^2^Department of Paediatrics, Maastricht University Medical Center, Maastricht, Netherlands; ^3^Department of Drug Delivery, Helmholtz Institute for Pharmaceutical Research Saarland, Saarbrücken, Germany

**Keywords:** surfactant therapy, dose response, respiratory distress syndrome, Portactant alfa, Bovactant, animal models

## Abstract

**Background:**

Poractant alfa (Curosurf^®^) and Bovactant (Alveofact^®^) are two animal-derived pulmonary surfactants preparations approved for the treatment of neonatal respiratory distress syndrome (nRDS). They differ in their source, composition, pharmaceutical form, and clinical dose. How much these differences affect the acute pulmonary response to treatment is unknown.

**Objectives:**

Comparing these two surfactant preparations in two different animal models of respiratory distress focusing on the short-term response to treatment.

**Methods:**

Poractant alfa and Bovactant were administered in a 50–200 mg/kg dose range to surfactant-depleted adult rabbits with acute respiratory distress syndrome induced by lavage and to preterm lambs (127–129 days gestational age) with nRDS induced by developmental immaturity. The acute impact of surfactant therapy on gas exchange and pulmonary mechanics was assessed for 1 h in surfactant-depleted rabbits and for 3 h in preterm lambs.

**Results:**

Overall, treatment with Bovactant 50 mg/kg or Poractant alfa 50 mg/kg did not achieve full recovery of the rabbits’ respiratory conditions, as indicated by significantly lower arterial oxygenation and carbon dioxide values. By contrast, the two approved doses for clinical use of Poractant alfa (100 and 200 mg/kg) achieved a rapid and sustained recovery in both animal models. The comparison of the ventilation indices of the licensed doses of Bovactant (50 mg/kg) and Poractant alfa (100 mg/kg) showed a superior performance of the latter preparation in both animal models. At equal phospholipid doses, Poractant alfa was superior to Bovactant in terms of arterial oxygenation in both animal models. In preterm lambs, surfactant replacement therapy with Poractant alfa at either 100 or 200 mg/kg was associated with significantly higher lung gas volumes compared to Bovactant treatment with 100 mg/kg.

**Conclusion:**

At the licensed doses, the acute pulmonary response to Poractant alfa was significantly better than the one observed after Bovactant treatment, either at 50 or at 100 mg/kg dose, in two animal models of pulmonary failure.

## Introduction

Neonatal respiratory distress syndrome (nRDS) is a major life-threatening consequence of preterm birth. The lungs of preterm infants developing nRDS are morphologically as well as biochemically immature and tend to collapse at end-expiration due to high intrapulmonary surface tension, arising from a primary lack of pulmonary surfactant (1). Pulmonary surfactant is a complex mixture of phospholipids and proteins that lines the alveolar surface and modulates the surface tension throughout the respiratory cycle (2). The synthesis, secretion, and intrapulmonary accumulation of pulmonary surfactant, however, only start at the boundaries between the canalicular and saccular phases of intra-uterine lung development, around the 23–25th weeks of gestation (3). Consequently, the incidence as well as the severity of nRDS is higher in infants born at a lower gestational age (GA) (4).

The development of surfactant replacement therapy, which consists of delivering an intratracheal bolus of exogenous surfactant directly to the lungs, dramatically changed the clinical management of RDS, thereby reducing the morbidity and the mortality associated with the disease (5). For over three decades, animal-derived surfactant preparations, principally from bovine or porcine origin, have been used for the prevention and treatment of nRDS (5–7).

Several clinical studies have attempted to determine the most efficacious surfactant preparation as well as the most advantageous dose and timing of administration (8–13). To maximize its effects, surfactant replacement therapy should be administered as early as possible in the course of nRDS (14). A combined analysis of these clinical trials revealed that the treatment with a higher phospholipid dose, as licensed for Poractant alfa (200 mg/kg), significantly reduces the mortality associated to nRDS compared to other surfactant preparations administered at lower doses such as Beractant (100 mg/kg) and Calfactant (105 mg/kg) (15, 16).

The comparison of the efficacy of Bovactant and Poractant alfa, two clinically available surfactant preparations, has not led yet to conclusive and univocal recommendations in the clinical arena (8, 15, 17, 18). Differences between Poractant alfa and Bovactant include the animal source (porcine vs. bovine), the extraction method (minced lung vs. lung wash), the pharmaceutical form (suspension vs. powder), the phospholipid and protein concentration and composition (a detailed description is available in Section “Surfactant Preparations”), and their licensed doses (100 or 200 mg/kg for Poractant alfa vs. 50 mg/kg for Bovactant) (14). Although clinically relevant outcomes must necessarily be addressed in controlled trials, the acute response to surfactant therapy in terms of gas exchange and lung mechanics can be better studied in appropriate preclinical animal models of surfactant deficiency such as surfactant-depleted rabbits (19–21) or preterm lambs with a primary surfactant deficiency (22–26).

Considering the licensed doses of Poractant alfa and Bovactant, we hypothesize a superior performance of the former preparation in terms of oxygenation and lung mechanics due to a higher phospholipid dose. To test this hypothesis, we performed an *in vivo* study using two well-characterized animal models: we first compared the performance of both preparations in surfactant-depleted adult rabbits with acute respiratory distress syndrome (ARDS) and, thereafter, in preterm lambs with primary nRDS. Furthermore, we also performed a head-to-head comparison between surfactant preparations delivered at an equal phospholipid dose.

## Materials and Methods

### Surfactant Preparations

Poractant alfa (Curosurf^®^, Chiesi Farmaceutici, Parma, Italy) is a natural surfactant, prepared from porcine lungs, containing almost exclusively polar lipids, in particular PC (about 70% of the total phospholipid content), and about 1% of specific low molecular weight hydrophobic proteins SP-B and SP-C, at a phospholipid concentration of 80 mg/ml.

Bovactant (Alveofact^®^, Lyomark Pharma, Oberhaching, Germany) is a natural surfactant prepared from lavaged bovine lungs, containing polar lipids and about 1% of specific low molecular weight hydrophobic proteins SP-B and SP-C, at a phospholipid concentration of 41.7 mg/ml. It is formulated as a lyophilized powder to be suspended right before its use.

### *In Vivo* Studies

The experimental protocols complied with all the European regulations regarding animal care and handling. The rabbit study complied with the Italian regulations for animal care and was approved by the local Ethics committee for animal welfare. The preterm lamb study complied with the Institutional Animal Ethics Committee of Maastricht University, The Netherlands (DEC 2011-097).

#### Surfactant-Depleted Adult Rabbit Model

The study was conducted in New Zealand white rabbits. Anesthesia was induced with intramuscular ketamine 20 mg/kg combined with 5 mg/kg xylazine and was maintained by continuous infusion of ketamine (20 mg/kg/h). Animals were then tracheotomized and catheters were inserted into the right jugular vein for fluid infusion and into the right carotid artery for blood sampling and monitoring. Paralysis was induced with pipecuronium bromide (0.3 mg/kg/30 min intravenously, i.v.). Mechanical ventilation was provided with a pressure-controlled ventilator (Fabian HFO, Acutronic, Zug, Switzerland) with the following initial settings: respiratory rate (RR) of 40 breaths/min, inspiratory time of 0.5 s, fraction of inspired oxygen (FiO_2_) 1.0, positive-end expiratory pressure (PEEP) of 3 cmH_2_O. Ventilation settings were modified accordingly to maintain a target tidal volume (*V*_T_) between 7 and 9 ml/kg with the peak inspiratory pressure (PIP) not exceeding 15 cmH_2_O.

The lungs of the animals were subjected to repeated bronchoalveolar lavages (BALs) to withdraw the intrapulmonary surfactant pool, as described by Ricci et al. (21). Briefly, BALs were performed by flushing the airways with 30 ml of pre-warmed 0.9% NaCl solution, followed by a short recovery period in-between, until an arterial oxygen partial pressure (PaO_2_) value <150 mmHg was reached. Then, if after 15 min of stabilization on mechanical ventilation the respiratory distress was confirmed with a new blood gas analysis (PaO_2_ < 150 mmH, at FiO_2_ of 1.0), the animals were randomized to one of the treatment groups (*n* = 6 each): animals received an intratracheal bolus of (1) 200 mg/kg of Poractant alfa, (2) 100 mg/kg of Poractant alfa, (3) 50 mg/kg of Poractant alfa, (4) 100 mg/kg of Bovactant, (5) 50 mg/kg of Bovactant, or (6) no surfactant, just mechanical ventilation (control group). Following surfactant administration, animals were maintained in mechanical ventilation for 60 min. Arterial blood samples were taken at baseline (before BALs), after surfactant depletion, and then 5, 30, and 60 min following surfactant treatment. Dynamic compliance (*C*_dyn_) was registered at the same intervals.

#### Preterm Lamb Model with Primary Surfactant Deficiency

Texel sheep were time-mated to yield animals of appropriate GA. Repeated antenatal ultrasound scans insured correct GA and adequate *in utero* growth. Preterm lambs were operatively delivered at 127–129 days GA (term 150 days). At this GA, lung maturation resembles approximately 27–29 weeks of gestation in humans. The ewes were sedated with thiopental i.v. (0.5–1.0 g/50 kg), intubated, and mechanically ventilated. Sedation was maintained with continuous inhalation of isoflurane (2%), and guided by depth of sedation and i.v. remifentanil (0.75 µg/kg/min) for pain management. C-section was performed as a modified EXIT procedure as described before (23). Umbilical artery and vein were catheterized while the lambs remained on perfusion *via* the placenta. After cutting the umbilical cord, the animals were weighed and moved to a warmer bed (CosyCot, Fisher&Paykel, Auckland, New Zealand) with a Babylog 8000 mechanical ventilation device (Dräger, Lübeck, Germany).

Lambs were sedated *via* the umbilical line with midazolam and ketamine, and orally intubated with a cuffed tube. Lambs were randomized to one of four treatment groups: Poractant alfa 100 mg/kg (*n* = 7), Poractant alfa 200 mg/kg (*n* = 6), Bovactant 50 mg/kg (*n* = 9), or Bovactant 100 mg/kg (*n* = 8). A series of *n* = 3 animals were given Bovactant 200 mg/kg. Surfactant was administered endotracheally prior to connection to the ventilator. Animals were afterward ventilated for 3 h (initial settings PIP 30 cmH_2_O, PEEP 8 cmH_2_O, RR 60/min); FiO_2_ 1.0, inspiratory to expiratory ratio (*I*:*E*) 1:2. During ventilation, regular blood gas analyses were performed and PIP was adjusted to maintain an arterial carbon dioxide partial pressure (PaCO_2_) between 45 and 60 mmHg. Primary readout was oxygenation measured as the PaO_2_ in the course of the 3-h ventilation. At the end of the experiment, the animals were euthanized. Directly after euthanasia, lambs were disconnected from ventilation and the thorax was opened. A post-mortem pressure–volume (*P*/*V*) curve was performed. The lung was inflated to 40 cmH_2_O and afterward passively deflated *via* the endotracheal tube connected to a manometer. Corresponding gas volumes were recorded and afterwards adjusted for body weight as previously described (23).

### Ventilation Indices

To compare the performance of Poractant alfa versus Bovactant, the gas exchange data and the ventilation parameters were computed to obtain the Ventilation Efficiency Index (VEI) and the Oxygenation Index (OI) (21). VEI was calculated to evaluate the overall ventilation efficiency of mechanically ventilated animals, independently from the ventilation settings:

(1) $$\text{VEI}=3,800/\left[\left(\text{PIP}/\text{PEEP}\right)*\text{RR}*{\text{PaCO}}_{2}\right].$$

The OI was calculated to describe the severity of pulmonary dysfunction in ventilated animals.

(2) $$\text{OI}={\text{FiO}}_{2}*\text{MAP}*100/{\text{PaO}}_{2},$$

where MAP is the mean airway pressure.

First, we compared the OI and VEI of the licensed doses of Bovactant (50 mg/kg) and the lowest licensed dose Poractant alfa (100 mg/kg). We further performed a head-to-head comparison of the VEI and OI of both surfactant preparations administered at the same phospholipid dose (100 mg/kg).

### Statistical Analysis

All data are presented as mean ± SEM. For the rabbit model, raw data were analyzed and compared by repeated measures two-way analysis of variance (ANOVA) as a function of group and time, followed by Tukey’s *t post hoc* test. Statistical analysis was performed using GraphPad software, version 6.0. For the lamb model, normally distributed data were compared using one-way ANOVA. Groups of interest were compared using Student’s *t*-test. Non-normally distributed data were compared using Kruskal–Wallis test, groups of interest were compared using Mann–Whitney test. Statistics were performed with IBM SPSS v20.

## Results

### Surfactant-Depleted Adult Rabbit Model

The acute pulmonary response to Poractant alfa and Bovactant was first evaluated in the lung-lavaged adult rabbit model by monitoring the arterial gases evolution after the administration of different doses of Bovactant (50 and 100 mg/kg) and Poractant alfa (50, 100, and 200 mg/kg). At baseline, before the BALs, neither the body weight nor the PaO_2_, or PaCO_2_ significantly differed between groups (Table S1 in Supplementary Material). Respiratory failure was successfully induced by repeated BALs, as indicated by a dramatic drop of PaO_2_ values from around 500 mmHg to values below 100 mmHg with a FiO_2_ of 1.0.

**Figure 1:**
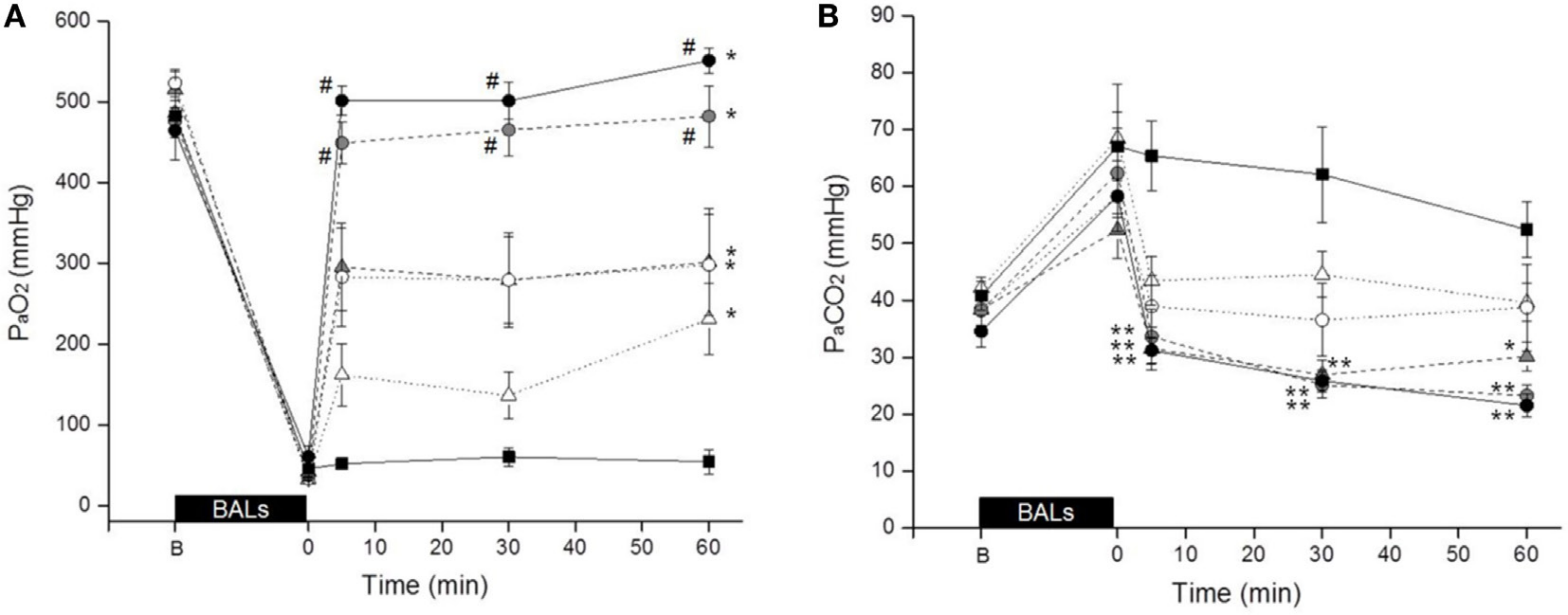
Mean PaO_2_ **(A)** and PaCO_2_ **(B)** values of surfactant-depleted rabbits treated with Poractant alfa at doses of 50 mg/kg (white circles), 100 mg/kg (gray circles), and 200 mg/kg (black circles) or with Bovactant at doses of 50 mg/kg (white triangles) and 100 mg/kg (gray triangles). An untreated group of animals managed with mechanical ventilation served as control (black squares). BALs, bronchoalveolar lavages; B, baseline, before BALs. Mean ± SEM is shown. *P vs. Control <0.05; **P vs. Control <0.01; and ^#^*P* vs. Bovactant (50 and 100 mg/kg) <0.05.

The mean PaO_2_ rapidly increased in all groups after surfactant treatment. Nevertheless, the increase of the PaO_2_ was dose dependent as well as preparation dependent. The highest mean PaO_2_ at any time interval was achieved by Poractant alfa administered at 200 mg/kg (Figure 1A). For this particular preparation, all tested doses induced a significant improvement of PaO_2_ compared to untreated control animals. No significant differences were observed between Poractant alfa instilled at 100 or 200 mg/kg, although the latter showed a slightly higher mean PaO_2_. However, these two doses showed a significant advantage over the 50 mg/kg dose of Poractant alfa.

With regard to Bovactant, a significant improvement of oxygenation compared to the control group could only be observed after administering the highest dose (100 mg/kg). The animals treated with the low Bovactant dose (50 mg/kg) showed a variable response to treatment: 5 min after surfactant administration, only two animals out of six showed PaO_2_ values higher than 200 mmHg, whereas the PaO_2_ values of the remaining four animals were below 120 mmHg (Figure S1 in Supplementary Material). In the case of Bovactant, the use of the higher dose (100 mg/kg) was associated with a significantly higher PaO_2_ at 5 and 30 min but not at 60 min compared to the animals treated with the low dose. The mean PaO_2_ values of Bovactant 100 mg/kg were comparable to those of Poractant alfa 50 mg/kg, but significantly lower than the ones achieved by Poractant alfa 100 and 200 mg/kg at any time interval.

The trends in terms of PaCO_2_ inversely correlated with the results obtained for PaO_2_ (Figure 1B). The administration of Poractant alfa 200 mg/kg, Poractant alfa 100 mg/kg, and Bovactant 100 mg/kg produced a significant decrease of the mean PaCO_2_ compared to the control group, which was just managed with mechanical ventilation. The mean PaCO_2_ values of the Poractant alfa and Bovactant 50 mg/kg groups did not differ significantly from untreated control animals.

**Figure 2:**
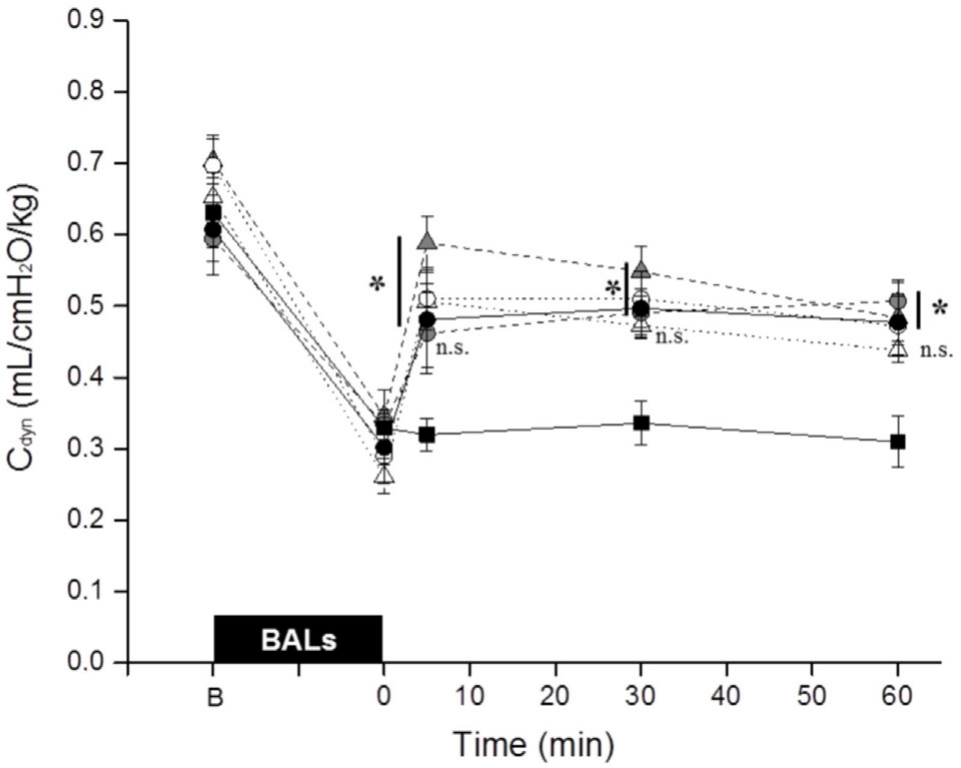
Mean dynamic compliance (*C*_dyn_) of surfactant-depleted rabbits treated with Poractant alfa at doses of 50 mg/kg (white circles), 100 mg/kg (gray circles), and 200 mg/kg (black circles) or with Bovactant at doses of 50 mg/kg (white triangles) and 100 mg/kg (gray triangles). An untreated group of animals managed with mechanical ventilation served as control (black squares). BALs, bronchoalveolar lavages; B, baseline, before BALs; n.s., not significant. Mean ± SEM is shown. *P vs. Control <0.05.

The BALs induced a dramatic drop of the *C*_dyn_ in all groups (Figure 2). Nevertheless, the values increased in all surfactant-treated groups immediately after surfactant administration. There were no statistical differences between surfactant-treated groups. All groups had a significantly higher *C*_dyn_ compared to untreated control animals except the Poractant alfa 100 mg/kg group at 5 min and the Bovactant 50 mg/kg group at 30 and 60 min.

### Preterm Lamb Model with Primary Surfactant Deficiency

In a second *in vivo* experimental session, the performance of Poractant alfa and Bovactant was evaluated in preterm lambs. The experimental design of this session included two animal groups of Poractant alfa treated with 100 or 200 mg/kg (both licensed doses), and two additional groups of Bovactant treated with the licensed dose, 50 mg/kg, or alternatively with a dose of 100 mg/kg. Differently from the rabbit session, it was decided here to drop the Poractant alfa 50 mg/kg treatment and insert the group of Bovactant 200 mg/kg for attempting a comparison with the Poractant alfa 200 mg/kg treatment. Unfortunately, this could not be performed because endotracheal administration of the necessary volume led to tube obstruction, afflux, and loss of not quantifiable amounts of surfactant, which made comparison and management impossible.

**Figure 3:**
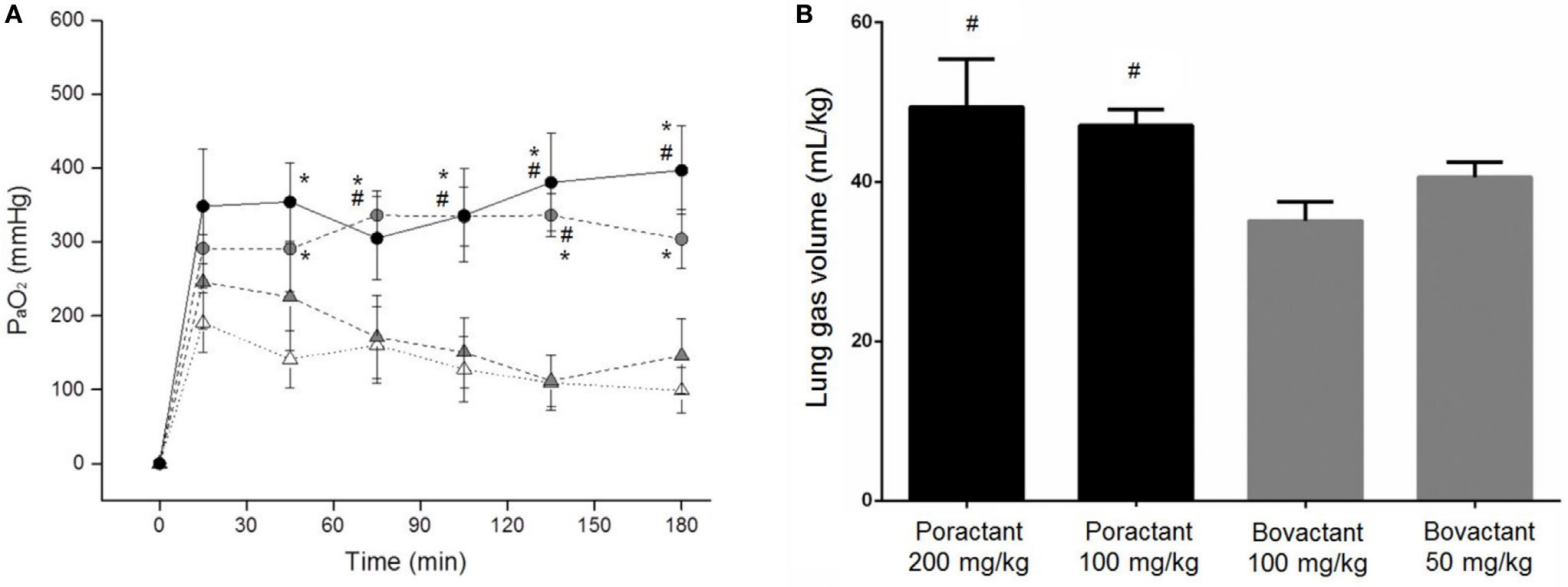
**(A)** Mean PaO_2_ values of preterm lambs treated with Poractant alfa at doses of 100 mg/kg (gray circles) and 200 mg/kg (black circles) or with Bovactant at doses of 50 mg/kg (white triangles) and 100 mg/kg (gray triangles). **(B)** Post-mortem gas volumes of preterm lambs treated with Poractant alfa or with Bovactant. Mean ± SEM is shown. *P vs. Bovactant 50 mg/kg <0.05; ^#^*P* vs. Bovactant 100 mg/kg <0.05.

Lambs among groups did not significantly differ either in birth weight or in pH values at baseline (Table S2 in Supplementary Material). We registered a significantly higher mean PaO_2_ in animals treated with Poractant alfa, regardless of the dose, compared to animals treated with Bovactant 50 mg/kg at 45 min after surfactant treatment until the end of the experimental period (Figure 3A). Oxygenation was as well significantly higher in animals treated with Poractant alfa 100 or 200 mg/kg than in animals treated with Bovactant 100 mg/kg from 75 to 135 min, although at 180 min, only the mean PaO_2_ of Poractant alfa administered at a dose of 200 mg/kg was significantly higher compared to Bovactant 100 mg/kg. No significant differences in oxygenation were observed between different doses of the same surfactant preparation.

The lung mechanics of the preterm lambs were investigated *post-mortem* by performing a *P*/*V* curve. The intrapulmonary gas volumes determined after inflating the lungs to a pressure of 40 cmH_2_O were higher in Poractant alfa-treated animals compared to Bovactant-treated animals. Significant differences were detected between Poractant alfa (irrespective of the administered dose) and the group of animals treated with a dose of 100 mg/kg of Bovactant (Figure 3B).

### Ventilation Indices

Bovactant is licensed to be administered at a dose of 50 mg/kg, whereas Poractant alfa is licensed to be administered at either 100 or 200 mg/kg (14). In order to perform a comparison between the licensed doses of both preparations, we calculated the VEI and OI of the rabbits and lambs treated with 50 mg/kg of Bovactant and 100 mg/kg of Poractant alfa, the lowest licensed dose for this surfactant preparation.

**Figure 4:**
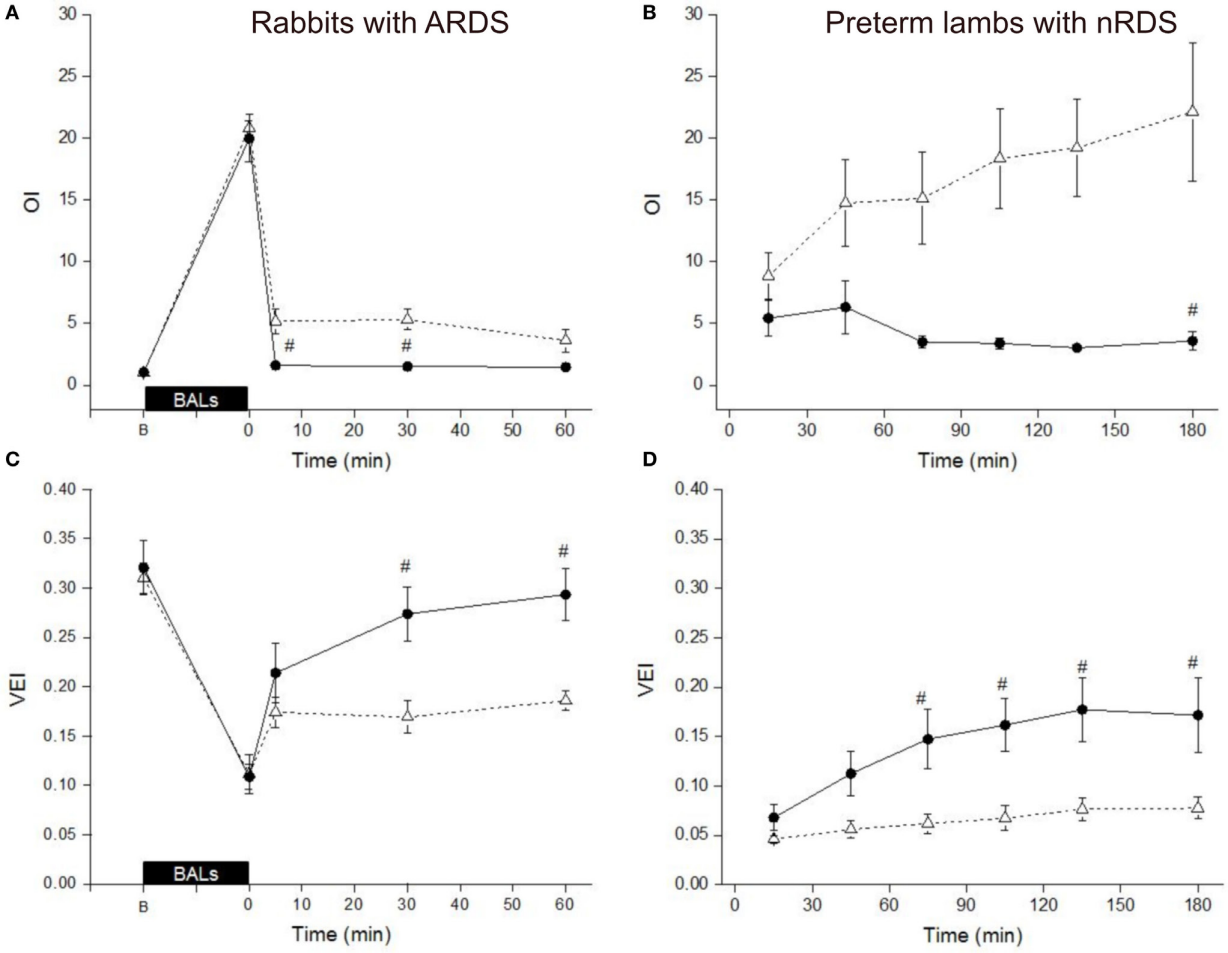
Mean Oxygenation Index (OI) values **(A,B)** and Ventilation Efficiency Index (VEI) values **(C,D)** of surfactant-depleted rabbits **(A,C)** and preterm lambs **(B,D)** treated with Poractant alfa at a dose of 100 mg/kg (black circles) or with Bovactant at a dose of 50 mg/kg (white triangles). BALs, bronchoalveolar lavages; B, baseline, before BALs. Mean ± SEM is shown. ^#^*P* vs. Bovactant 50 mg/kg <0.05.

In surfactant-depleted rabbits, the BALs produced an abrupt impairment of ventilation as shown by a dramatic increase of the OI as well as a pronounced drop of the VEI in both groups (Figures 4A,C). Treatment of the rabbits with Poractant alfa (100 mg/kg) rapidly restored the mean OI to baseline values, already at 5 min. Treatment of the rabbits with Bovactant (50 mg/kg) improved the OI values but to a lesser extent than Poractant alfa, which accounted for significantly better OI values favoring Poractant alfa at 5 and 30 min following surfactant administration. Similarly, the degree of improvement of the VEI in the rabbit model was also higher in the Poractant alfa group than in the Bovactant group, reaching significant differences between groups at 30 and 60 min.

In preterm lambs, the mean OI values could be kept rather low following Poractant alfa 100 mg/kg administration. On the contrary, the mean OI increased in the lamb group treated with Bovactant 50 mg/kg, with a significant difference between the groups at 180 min (Figure 4B). The mean VEI gradually increased over the first 2 h in the group of lambs treated with Poractant alfa 100 mg/kg. However, such an improvement could not be found if the lambs that were treated with Bovactant 50 mg/kg. The mean VEI was significantly better in the Poractant alfa 100 mg/kg group compared to the Bovactant 50 mg/kg group at 75, 105, 135, and 180 min (Figure 4D).

**Figure 5:**
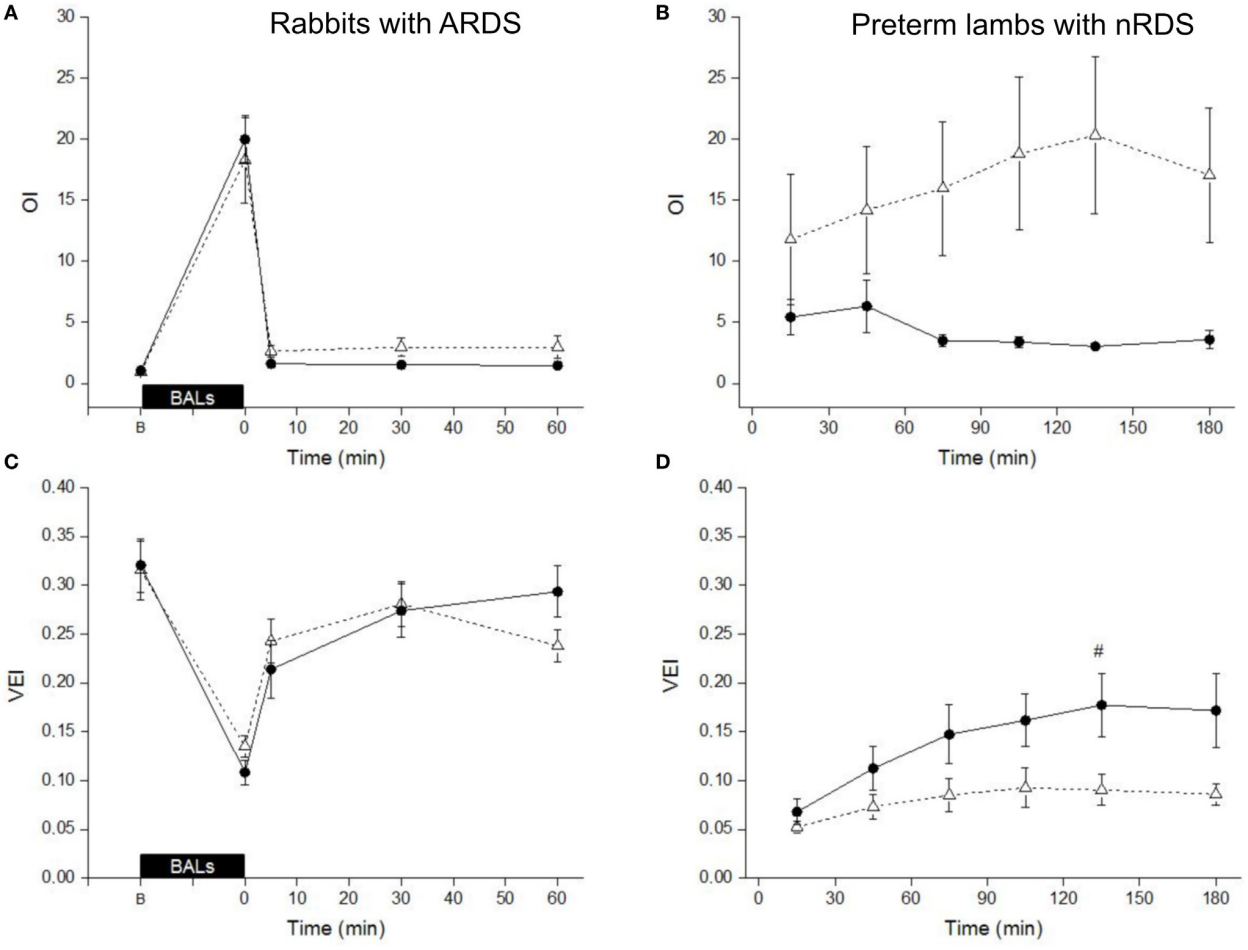
Head-to-head comparison of the mean Oxygenation Index (OI) values **(A,B)** and Ventilation Efficiency Index (VEI) values **(C,D)** of surfactant-depleted rabbits **(A,C)** and preterm lambs **(B,D)** treated with Poractant alfa at a dose of 100 mg/kg (black circles) or with Bovactant at a dose of 100 mg/kg (white triangles). BALs, bronchoalveolar lavages; B, baseline, before BALs. Mean ± SEM is shown. ^#^*P* vs. Bovactant 50 mg/kg <0.05.

The head-to-head comparison between Bovactant and Poractant alfa administered at 100 mg/kg did not show significant differences with regards to OI neither in surfactant-depleted rabbits nor in preterm lambs despite a tendency to lower OI values in animals treated with Poractant alfa (Figures 5A,B). Moreover, the pulmonary response to Poractant alfa was uniform in preterm lambs and showed a downward trend throughout the observational period. Conversely, the pulmonary response to Bovactant was not uniform and the mean OI worsened slightly over the 3 h of follow-up. No significant differences were detected in terms of VEI between surfactant-depleted rabbits treated with Bovactant or with Poractant alfa at 100 mg/kg. However, preterm lambs treated with Poractant alfa had higher mean VEI values than Bovactant-treated lambs reaching statistical significance at the 135-min time-point (Figures 5C,D).

## Discussion

In the present work, we have compared the acute pulmonary response to Poractant alfa and Bovactant administered at different doses in two different animal models: surfactant-depleted adult rabbits with ARDS and preterm lambs with primary nRDS. Among all listed natural surfactant preparations, we chose Bovactant and Poractant alfa for this comparison since there is little information on a head-to-head comparison in the literature. Given the literature for both surfactant preparations in different clinical and experimental settings (5, 9, 27–35), we have chosen these two preparations for our experimental study not only in different models of respiratory failure but also tested equal doses outside the clinical recommendations of the producers. Irrespective of the dose and preparation, surfactant administration improved the overall pulmonary status of surfactant-depleted rabbits. However, the intratracheal administration of Poractant alfa at 100 or 200 mg/kg was associated with a significantly higher oxygenation in comparison to Bovactant at either 50 or 100 mg/kg in both animal models. Moreover, the lung gas volumes of the lambs treated with Poractant alfa were significantly higher than the lung gas volumes of the lambs treated with Bovactant at a dose of 100 mg/kg. We further performed a comparison of the OI and the VEI of the licensed dose for Bovactant (50 mg/kg) and the lowest licensed dose for Poractant alfa (100 mg/kg), and we found a superior performance of the latter preparation in surfactant-depleted rabbits as well as in preterm lambs.

The proof of concept for an effective surfactant replacement therapy was first described in the early seventies by Enhörning and Robertson (36). They demonstrated a significant improvement of lung mechanics in premature rabbit pups after pharyngeal deposition of natural surfactant extracts. More than 40 years later, surfactant replacement therapy with natural surfactant extracts remains as a life-saving treatment for preterm infants with nRDS (14). So far, it was demonstrated that animal-derived surfactants are more effective than protein-free synthetic surfactants (37) and are still recommended over surfactant preparations containing a single SP analog (14). Although at variable fractions, all natural surfactants contain SP-B and SP-C. A higher efficacy of natural surfactants in comparison to the single-peptide synthetic preparations has been attributed to the presence of these small-hydrophobic proteins (38). In this regard, the new generation synthetic surfactant CHF5633 contains SP-B and SP-C analogs and has shown an equivalent efficacy to natural surfactants and a higher resistance to inactivation in various *in vivo* models (23, 25, 39, 40). CHF5633 is currently being tested in human preterm neonates (41).

Clinically available natural surfactant preparations differ in the animal source, the extraction methods, the phospholipid concentration, and the licensed dose. Several clinical trials have attempted to elucidate the most effective natural surfactant preparation and the most appropriate surfactant dose (8–13). Retrospective studies of these trials indicate that Poractant alfa significantly reduced the mortality compared to Beractant or Calfactant administered at doses of 100 and 105 mg/kg, respectively, provided that Poractant alfa was administered at a dose of 200 mg/kg (15, 16). Unfortunately, the scarce clinical trials comparing Bovactant and Poractant alfa are inconclusive and of a relatively low sample size to draw a definitive conclusion (8, 17, 18). Moreover, to date, no preclinical data were available which directly compares the acute effects on the overall lung function of these two surfactant preparations, which this study provides.

In the present study, we have compared the acute pulmonary effects of Bovactant and Poractant in surfactant-depleted rabbits and in preterm lambs. Despite not being a nRDS model, the lung-lavaged adult rabbit is a reproducible model of ARDS (19, 20). On the other hand, the preterm lamb model with a primary nRDS is the gold standard model to study the efficacy of exogenous surfactant preparations (22–26, 40). In both *in vivo* models, Poractant alfa administered at either 100 or 200 mg/kg was superior to Bovactant administered at 50 or 100 mg/kg in terms of arterial oxygenation. We included in our study a group of surfactant-depleted rabbits treated with Poractant alfa at a dose of 50 mg/kg to match the licensed dose of Bovactant. At this low phospholipid dose, both surfactant preparations improved the *C*_dyn_ of surfactant-depleted rabbits immediately after administration. This increase of *C*_dyn_ is in good agreement with the study conducted by Ikegami et al. in preterm lambs in which doses of at least 50 mg/kg were necessary to return *P*/*V* curves to normal values (42). Nevertheless, a clear increase of PaO_2_ as well as a significantly lower PaCO_2_ was observed in surfactant-depleted rabbits by doubling the surfactant dose from 50 to 100 mg/kg, irrespective of the surfactant preparation. This fact strongly suggests that the optimal phospholipid dose for surfactant replacement therapy should not be lower than 100 mg/kg. In line with this observation, Gortner et al. reported a significantly higher PaO_2_/FiO_2_ and reduced lung barotrauma in preterm babies (24–29 weeks GA) treated with a 100 mg/kg dose of Bovactant (*n* = 42) compared to the low dose (50 mg/kg) infant-group (*n* = 48) (43). This study of Gortner et al. was investigator initiated and took into account the results from previous clinical studies, which showed a high failure rate after the manufacturer’s recommendation of 50 mg/kg (29).

The OI and the VEI of the animals treated with the licensed doses of Bovactant and Poractant alfa was computed in order to compare the efficacy of the surfactant preparation taking into consideration not only the PaO_2_ and PaCO_2_ but also the ventilation parameters. If the licensed doses are considered, the OI as well as the VEI scores were significantly better for Poractant alfa compared to Bovactant in both animal models. The comparison between surfactant preparations at the same phospholipid dose yielded different results. In surfactant-depleted rabbits with ARDS, there were no differences between surfactant preparations, indicating once again the benefits of a starting surfactant dose of 100 mg/kg for Bovactant. In preterm lambs with primary nRDS, the trends toward a gradual improvement of OI and VEI were apparent. On the other hand, in the preterm lambs treated with Bovactant, regardless of the dose, the VEI was rather low and steady and the OI gradually worsened over the follow-up period. Hence, in the setting of a more severe respiratory distress, Poractant alfa performed significantly better than Bovactant. This finding can be considered of clinical relevance since current trends in the treatment of nRDS focus primarily on the use of non-invasive ventilation treatments for mild nRDS and surfactant replacement therapy for moderate-to-severe nRDS.

In addition to the differences on gas exchange, we also registered significantly higher lung gas volumes in preterm lambs treated with 100 mg/kg of Poractant alfa compared to lambs treated with Bovactant at the same dose. These differences in performance between preparations administered at the same dose might be related to differences in composition. Poractant alfa exhibits a higher amount of plasmalogens and polyunsaturated fatty acid-containing phospholipids, which in turn contribute to improve the surface activity of lipid mixtures (30). This slight difference in composition between preparations might have a significant impact on the biophysical properties and in their sensitivity toward inhibition by plasma proteins such as fibrinogen, albumin, and hemoglobin (44). The biophysical properties of Poractant alfa and Bovactant have been studied *in vitro* showing a faster and more stable film formation with Poractant alfa (45).

We would like to acknowledge that the overall response to surfactant replacement therapy in the clinical context of nRDS is extremely complex, multifactorial, and takes place in a significantly longer time scale than the observational period of the present study. However, our aim here was to compare the acute pulmonary response of the two surfactants under controlled conditions, when surfactant is more effective and its surface activity can be assessed in the absence of inflammation and/or edema, which may influence the pulmonary effect of surfactant instillation (46, 47). Unfortunately, we could not perform a direct comparison between Poractant alfa and Bovactant administered at 200 mg/kg in preterm lambs due to the large volume required for the intratracheal administration of the latter preparation. The restricted observational period, a common limitation of *in vivo* studies, might have been the reason why Poractant alfa 200 mg/kg did not differ from 100 mg/kg. Nevertheless, according to clinical evidence (15, 16), the use of the 200 mg/kg Poractant alfa dose should be considered in a clinical setting since it reduces mortality in the context of nRDS compared to other surfactants administered at 100 mg/kg and reduces the need for re-dosing.

## Ethics Statement

The experimental protocols complied with all the European regulations regarding animal care and handling. The rabbit study complied with the Italian regulations for animal care and was approved by the local Ethics committee for animal welfare. The preterm lamb study complied with the Institutional Animal Ethics Committee of Maastricht University, The Netherlands (DEC 2011-097).

## Author Contributions

FS, XM, MH, BK, and FB provided substantial contributions to the conception, design, and data interpretation of the work. FR, EK, DO, MN, MW, and TK took care of the acquisition and analysis of data for the work. All the authors contributed to the drafting the work and revising it critically for important intellectual content; all the authors provided a final approval of the version to be published and agreed to be accountable for all aspects of the work in ensuring that questions related to the accuracy or integrity of any part of the work are appropriately investigated and resolved.

## Conflict of Interest Statement

This study was funded by Chiesi Farmaceutici S.p.A. (Parma, Italy), which is the employer of authors FR, FS, and FB. The animal-derived surfactant Poractant alfa (Curosurf, 80 mg/ml) was also supplied by Chiesi Farmaceutici S.p.A. XM served as consultants for Chiesi Farmaceutici S.p.A. in this study.
