# Supplementary material for: In Vivo Evaluation of the Acute Pulmonary Response to Poractant Alfa and Bovactant Treatments in Lung-Lavaged Adult Rabbits and in Preterm Lambs with Respiratory Distress Syndrome
Source: Front Pediatr. 2017 Aug 31;5:186. doi: 10.3389/fped.2017.00186 (PMC5583171; doi:10.3389/fped.2017.00186)
Supplement: Supplementary file 1 [file Table_1.DOCX]

**Supplementary information (SI)**

|  | **Groups** | **BASAL** | **15 MIN ST** |
| --- | --- | --- | --- |
| **paO_2_ mmHg** | **Negative Control** | 419,7 ± 64,8 | 45,5 ± 6,0 |
|  | **Bovactant 100mg/kg** | 515,2 ± 22,4 | 41,9 ± 6,8 |
|  | **Bovactant 50mg/kg** | 458,9 ± 30 | 32,7 ± 1 |
|  | **Poractant alfa 200mg/kg** | 464,4 ± 36,5 | 60,8 ± 12,9 |
|  | **Poractant alfa 100mg/kg** | 476,13 ± 16,2 | 32,7 ± 4 |
|  | **Poractant alfa 50mg/kg** | 476,13 ± 16,2 | 33, ± 3,2 |
| **paCO_2_ mmHg** | **Negative Control** | 39,8 ± 2,3 | 62,4 ± 4,8 |
|  | **Bovactant 100mg/kg** | 38,4 ± 2,7 | 52,4 ± 4,9 |
|  | **Bovactant 50mg/kg** | 42,2 ± 1,9 | 68,4 ± 9,6 |
|  | **Poractant alfa 200mg/kg** | 34,5 ± 2,8 | 58,3 ± 3,1 |
|  | **Poractant alfa 100mg/kg** | 38,2 ± 2,5 | 62,3 ± 7,9 |
|  | **Poractant alfa 50mg/kg** | 38,5 ± 3,1 | 58,3 ± 6,2 |
| **Birth Weight Kg** | **Negative Control** | 2,3 ± 0,2 |  |
|  | **Bovactant 100mg/kg** | 2,3 ± 0,1 |  |
|  | **Bovactant 50mg/kg** | 2,2 ± 0,1 |  |
|  | **Poractant alfa 200mg/kg** | 2,2 ± 0,1 |  |
|  | **Poractant alfa 100mg/kg** | 2,4 ± 0,1 |  |
|  | **Poractant alfa 50mg/kg** | 2,3 ± 0,1 |  |

**Table S1. Weights and gas exchange values of surfactant- depleted rabbits at the Basal and after a post-injury stabilization period of 15 min**

Arterial carbon dioxide partial pressure (PaCO_2_) oxygen partial pressure (PaO_2_) and weight in animals managed just with Mechanical Ventilation (MV) (Negative control) and in animals treated with Poractant alfa or Bovactant at different doses. Gas exchange values were recorded upon intubation (Basal) and 15 minutes post-injury (15 min ST). All animals had similar weight and gas exchange at basal and 15 min ST (data given as Mean ± SEM).
