# Supplementary material for: In Vivo Evaluation of the Acute Pulmonary Response to Poractant Alfa and Bovactant Treatments in Lung-Lavaged Adult Rabbits and in Preterm Lambs with Respiratory Distress Syndrome
Source: Front Pediatr. 2017 Aug 31;5:186. doi: 10.3389/fped.2017.00186 (PMC5583171; doi:10.3389/fped.2017.00186)
Supplement: Supplementary file 2 [file Table_2.DOCX]

**Supplementary information (SI)**

|  | **Groups** | **BASAL** |
| --- | --- | --- |
| **Birth Weight Kg** | **Bovactant 100mg/kg** | 2,5 ± 0,2 |
|  | **Bovactant 50mg/kg** | 2,5 ± 0,2 |
|  | **Poractant alfa 200mg/kg** | 2,7 ± 0,1 |
|  | **Poractant alfa 100mg/kg** | 2,4 ± 0,1 |
| **Male : Female ratio** | **Bovactant 100mg/kg** | 4 : 4 |
|  | **Bovactant 50mg/kg** | 4 : 5 |
|  | **Poractant alfa 200mg/kg** | 4 : 2 |
|  | **Poractant alfa 100mg/kg** | 2 : 5 |
| **First pH** | **Bovactant 100mg/kg** | 7,1 ± 0,1 |
|  | **Bovactant 50mg/kg** | 7,0 ± 0,1 |
|  | **Poractant alfa 200mg/kg** | 7,1 ± 0,1 |
|  | **Poractant alfa 100mg/kg** | 7,1 ± 0,1 |

**Table S2. Weights, male:female ratio and pH values at birth of preterm lambs.** Weight, male:female ratio and first pH in animals randomly assigned to treatment with Poractant alfa or Bovactant at different doses. All groups did not differ significantly in baseline characteristics (data given as Mean ± SEM).
