# Supplementary material for: In Vivo Evaluation of the Acute Pulmonary Response to Poractant Alfa and Bovactant Treatments in Lung-Lavaged Adult Rabbits and in Preterm Lambs with Respiratory Distress Syndrome
Source: Front Pediatr. 2017 Aug 31;5:186. doi: 10.3389/fped.2017.00186 (PMC5583171; doi:10.3389/fped.2017.00186)
Supplement: Supplementary file 3 [file Image_1.PDF]

Supplementary information (SI)

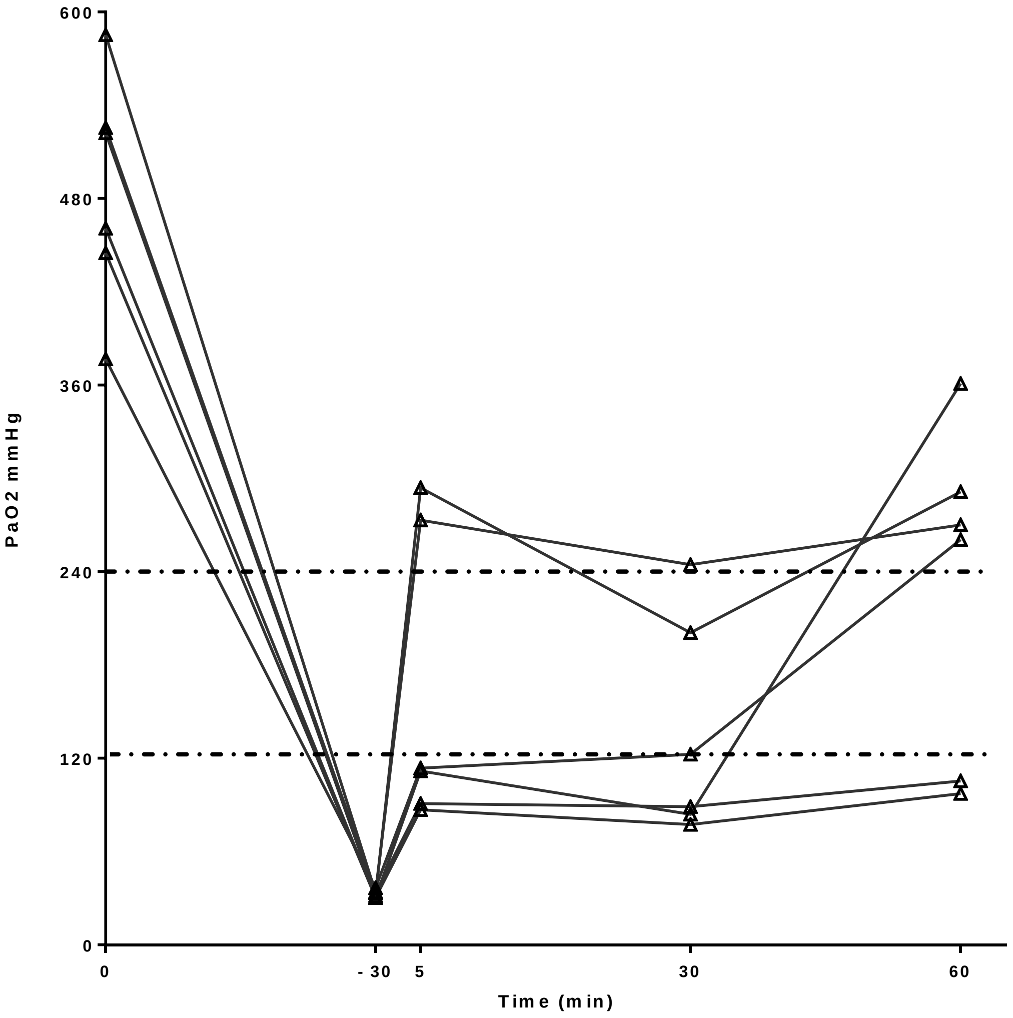

Figure S1 PaO_2 values of surfactant-depleted rabbits treated with Bovactant 50mg/kg

Mean PaO_2 values. Bovactant administered at a dose of 50mg/kg induced a variable response in terms of arterial oxygenation (PaO_2). At 5 minutes, only two animals showed a PaO_2 higher than 240 mmHg. The PaO_2 values of the remaining animals were lower than 120 mmHg.
